# Supplementary material for: A previously unrecognized membrane protein in the Rhodobacter sphaeroides LH1-RC photocomplex
Source: Nat Commun. 2021 Nov 2;12:6300. doi: 10.1038/s41467-021-26561-9 (PMC8564508; doi:10.1038/s41467-021-26561-9)
Supplement: Supplementary file 1 — Supplementary Information [file 41467_2021_26561_MOESM1_ESM.pdf]

SUPPLEMENTARY INFORMATION

A Previously Unrecognized Membrane Protein in the *Rhodobacter sphaeroides* LH1-RC Photocomplex

K. Tani, et al.

Supplementary Table 1 Cryo-EM data collection, refinement and validation statistics.

	LH1-RC-PufX-Protein-U complex (EMDB-31400, PDB ID: 7F0L)
**Data collection and processing**	
Magnification	92000
Voltage (kV)	200
Electron exposure (e–/Å^2)	42
Defocus range (μm)	–0.7 to –2.6
Pixel size (Å)	1.094
Symmetry imposed	C1
Initial particle images (no.)	551846
Final particle images (no.)	160488
Map resolution (Å)	2.9
FSC threshold	0.143
Map resolution range (Å)	313–2.9
**Refinement**	
Initial model used (PDB code)	5Y5S, 1PCR
Model resolution (Å)	3.1
FSC threshold	0.5
Model resolution range (Å)	140–2.9
Map sharpening *B* factor (Å^2)	–63
Model composition	
Non-hydrogen atoms	23458
Protein residues	2277
Ligands	112
B factors (Å^2)	
Protein	31.4
Ligand	33.3
R.m.s. deviations	
Bond lengths (Å)	0.007
Bond angles (°)	2.731
Validation	
MolProbity score	1.93
Clashscore	10.74
Poor rotamers (%)	1.93
Ramachandran plot	
Favored (%)	97.09
Allowed (%)	2.91
Disallowed (%)	0.00

Supplementary Table 2 Comparison of the distances of His–BChl(Mg) and BChl(Mg)–BChl(Mg) in LH1, LH2 and RC special pairs from various phototrophic bacteria.

LH1 or LH2	Distance of His(Nε2) to BChl–Mg (Å)[a]		Distance of Mg–Mg (Å)[a]	
	α	β	Long	Short
***Rba. sphaeroides* (LH1)**	**2.58**	**2.20**	**9.55**	**8.37**
Rsp. rubrum (LH1)	2.27	2.03	9.34	8.51
Rps. palustris (LH1-W)	2.93	2.71	9.61	8.29
Tch. tepidum (LH1)	2.19	2.19	8.88	8.72
Trv. strain 970 (LH1)	2.33	2.31	8.90	8.46
Blc. viridis (LH1)	2.54	2.25	8.8	8.5
Rfx. castenholzii (B880)	2.32	2.29	9.5	9.3
Rps. acidophila (B850)	2.34	2.34	9.5	8.8
Phs. molischianum (B850)	2.27	2.32	9.2	8.9

RC (special pair)	L-subunit	M-subunit	BChl *a* (L)–BChl *a* (M)
Rba. sphaeroides (LH1-RC)	**2.21**	**2.11**	**7.79**
Rba. sphaeroides (RC-only)	2.27	2.06	7.84
Rsp. rubrum	2.09	2.12	7.76
Rps. palustris	2.73	2.74	7.69
Tch. tepidum	2.17	2.19	7.87
Trv. strain 970	2.33	2.31	7.65
Blc. viridis	2.36	2.35	7.83

These values were derived from Protein Data Bank: 5Y5S for *Tch. tepidum*, 7C9R for *Trv.* strain 970, 6Z5S for *Rps. palustris*, 6ET5 for *Blc. viridis*, 5YQ7 for *Rfx. castenholzii*, 7EQD for *Rsp. rubrum*, 1NKZ for *Rps. acidophila*, 1LGH for *Phaeospirillum* (*Phs.*). *molischianum*, 2J8C for *Rba. sphaeroides* (RC-only).

Supplementary Table 3 Nucleotide sequences of the PCR primers used in the mutagenesis of protein-U (see Supplementary Fig. 7). The parts of ligation-tabs are underlined.

U-814F:	5’-TTGCATGCCTGCAGGTCCACCGCCACGAAGAGGAGGTTG
U-1R:	5’-GGTGCCTCCTTCAGATGCAAGC
U150F:	5’-TCTGAAGGAGGCACCGAACAGCAACTGACGGCACAGC
U+812R:	5’-GGGGATCCTCTAGAGTCGAGTTCACCGACTTCTTCGGCAAC

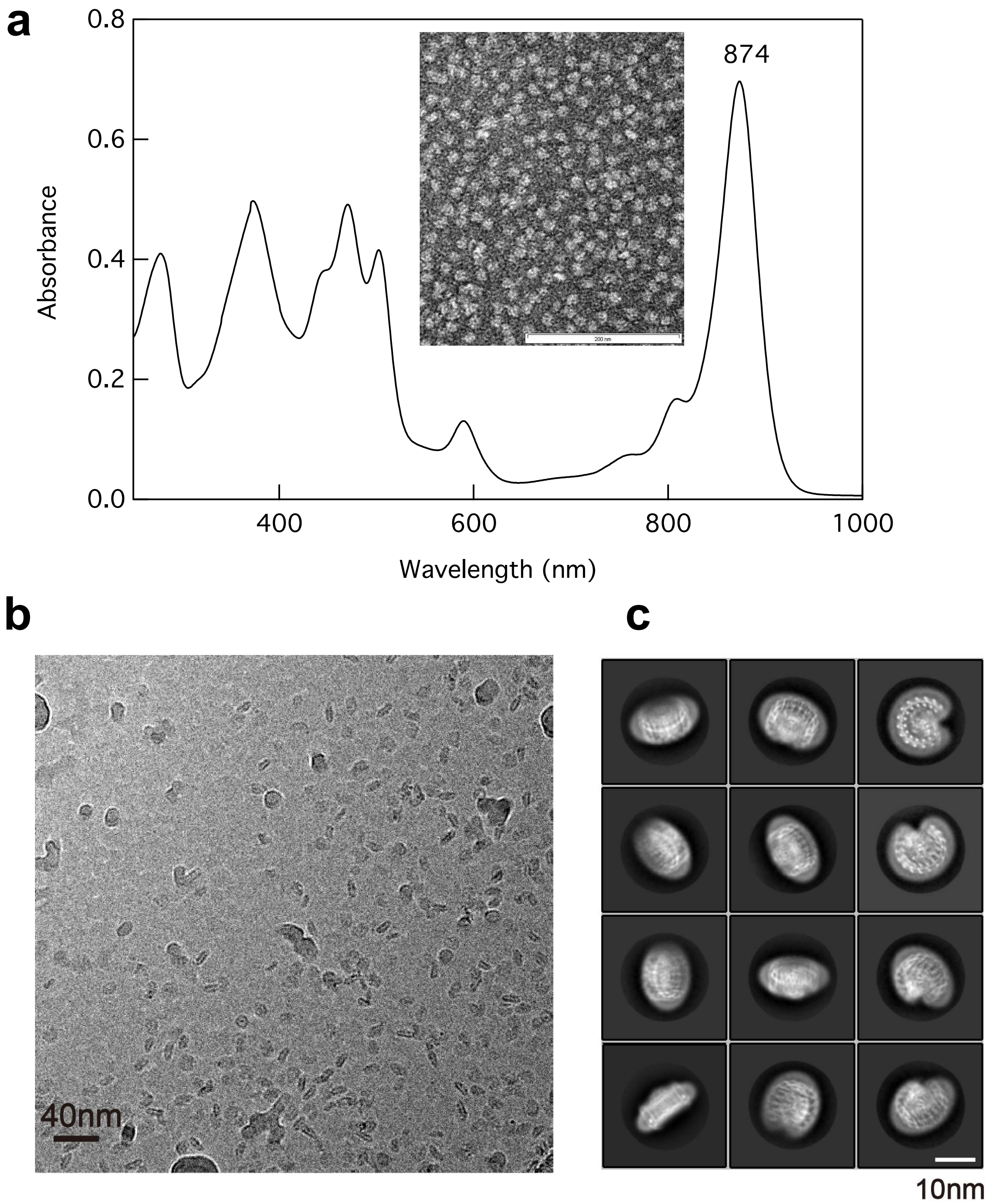

Supplementary Fig. 1 Absorption spectrum and cryo-EM of the *Rba. sphaeroides* IL106 monomeric LH1-RC complex. **(a)** Absorption spectrum of the purified monomeric LH1-RC at room temperature. Inset shows negatively stained LH1-RC particles obtained with 0.04 mg/mL LH1-RC in 20mM Tris-HCl (pH7.5) containing 0.05% DDM. Scale bar: 200 nm. **(b)** A representative cryo-EM micrograph. **(c)** Representative 2D class averages processed from the micrographs of LH1-RC.

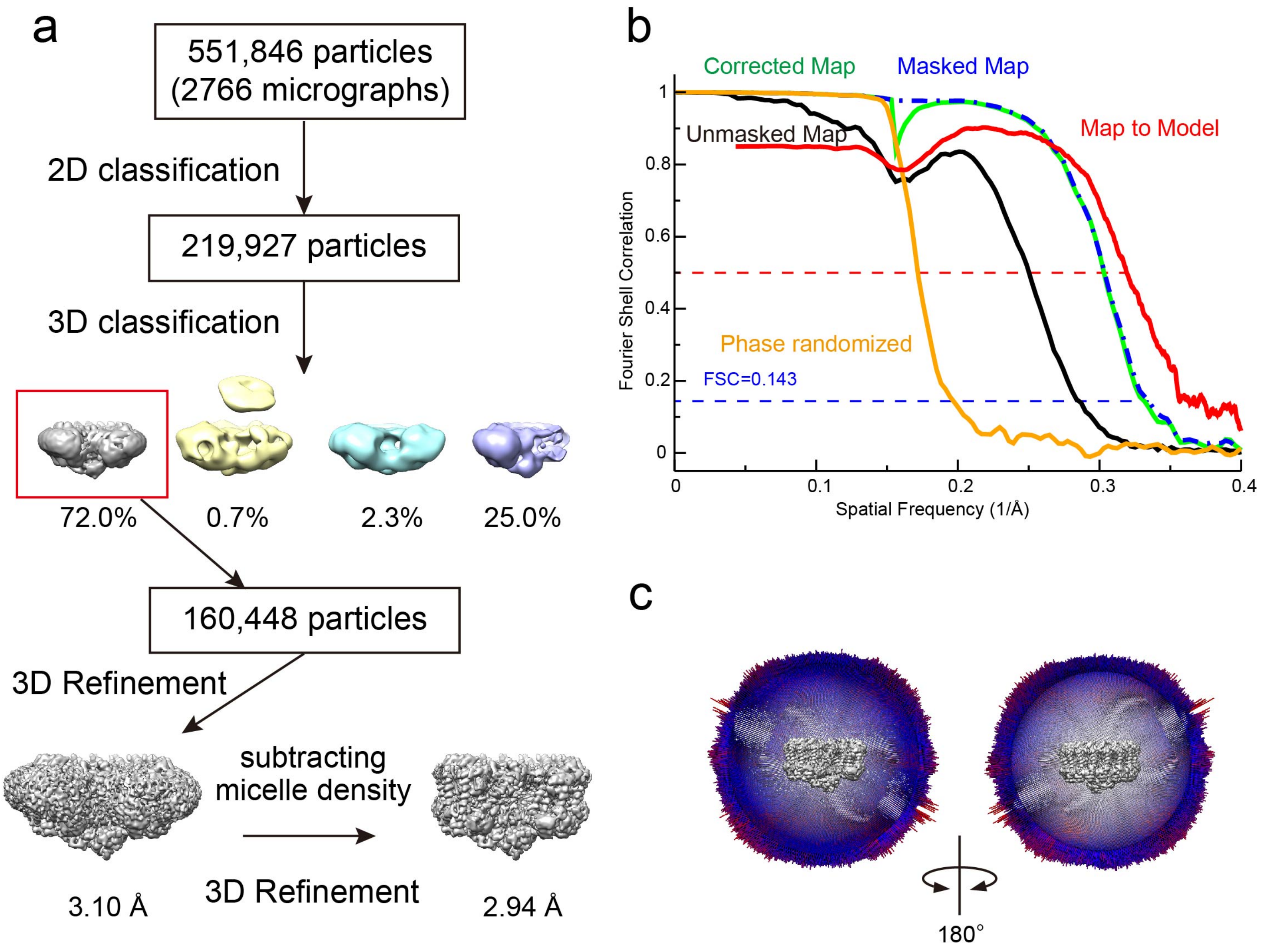

Supplementary Fig. 2 Structure determination of the *Rba. sphaeroides* IL106 monomeric LH1-RC complex. (a) Image processing flow of 3D classification and reconstruction. **(b)** The Fourier shell correlation (FSC) plot of the cryo-EM map (unmasked: black, masked: blue, phase randomized corrected: green, phase randomized: orange) and the FSC plot of the model versus the final map (red) are superimposed. **(c)** Angular distribution of reconstructed particles in the C1 map of LH1-RC complex. For clarity, the front half of angular distribution is removed.

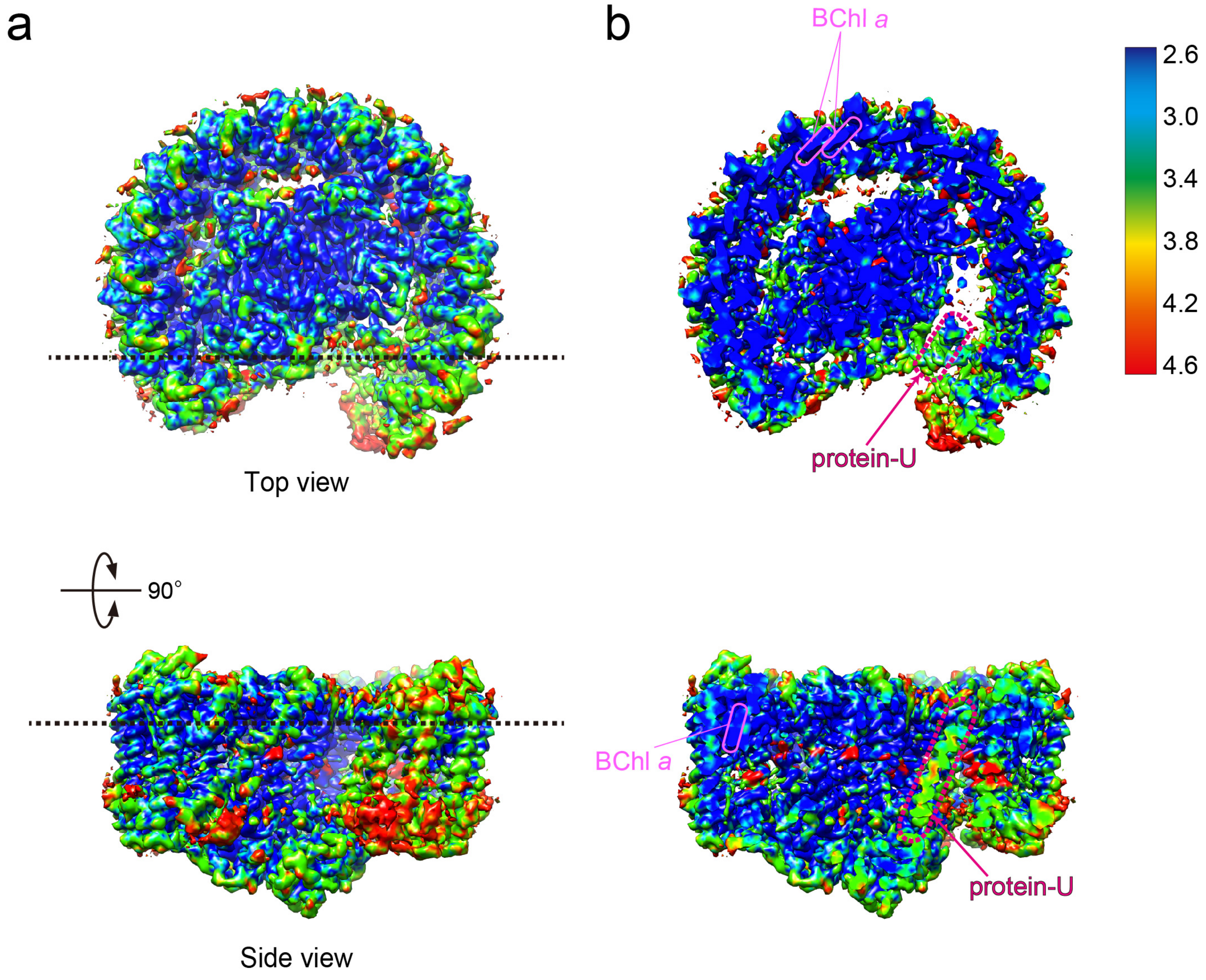

Supplementary Fig. 3 Local resolution representation of the structure of LH1-RC complex. (a) Top view from periplasmic side and side view parallel to the membrane plane. Each dotted line indicates the cross section line. **(b)** A central cross sectional view of the left side of the panel. A region indicated by circular pink line and magenta dotted line corresponds to BChl *a* and protein-U, respectively. The map is shown in the colors of the rainbow according to the estimated resolution from 4.6 Å (red) to 2.6 Å (blue).

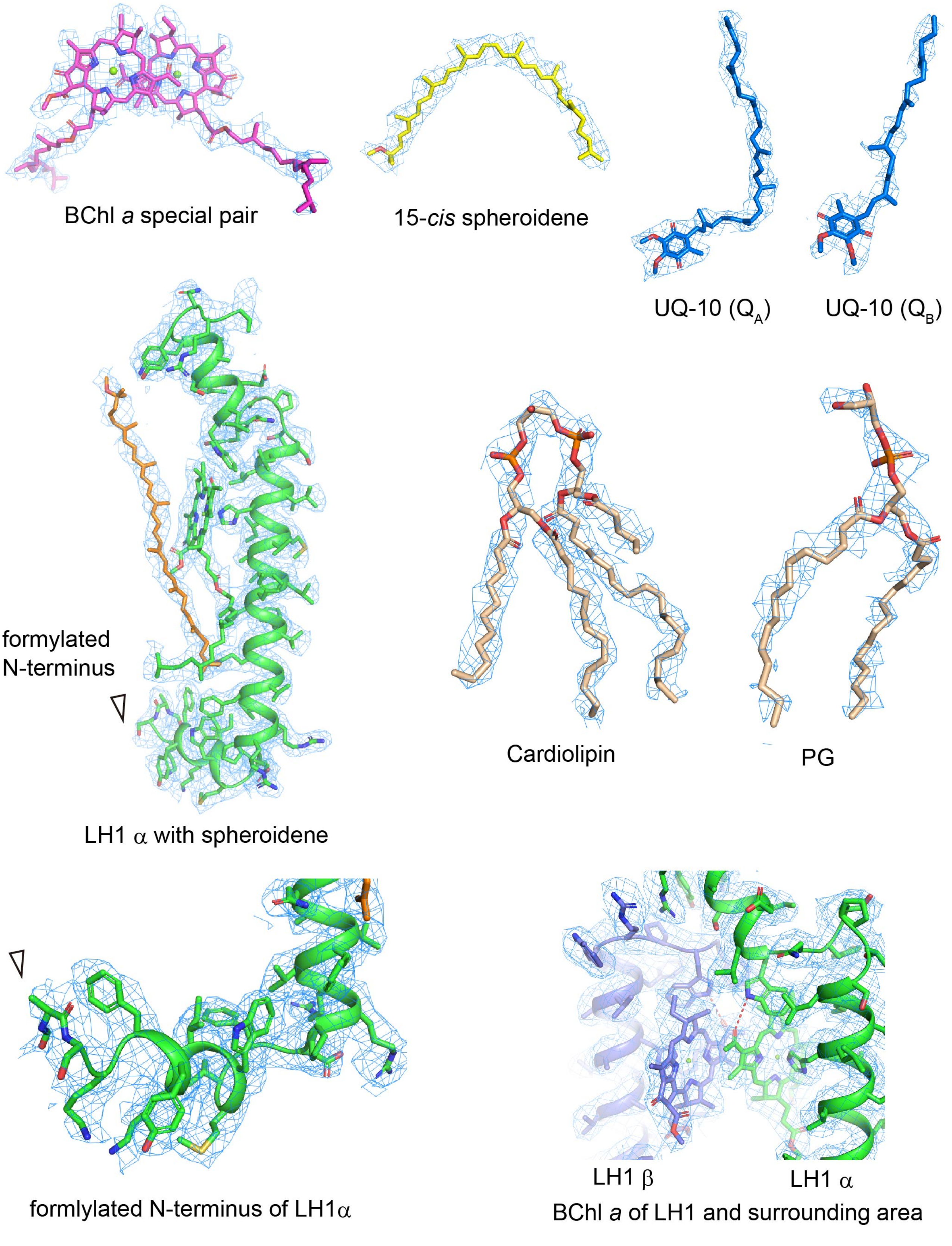

Supplementary Fig. 4 Cryo-EM densities and structural models in the the *Rba. sphaeroides* IL106 LH1-RC complex. The color codes of polypeptides are the same as in Fig. 1. The density maps are shown at a contour level of 3.0σ.

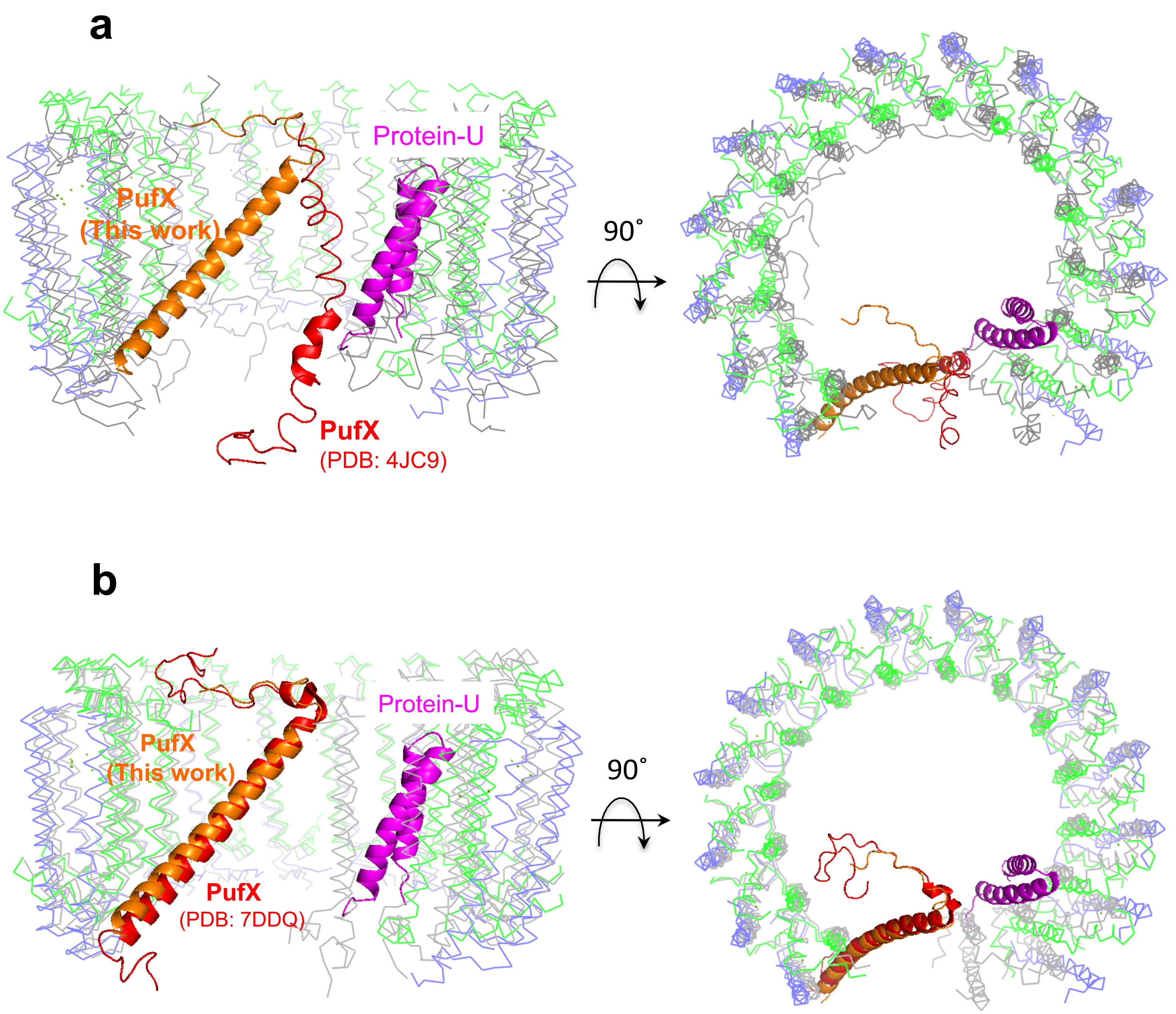

Supplementary Fig. 5 Comparison of the PufX structure determined in this work (orange cartoon) with those reported in other LH1-RC-PufX structures (red cartoon). **(a)** Side and top views of superposition of the Cα carbons of the LH1 αβ-polypeptides between the structure in this work (LH1-α, green ribbon; LH1-β, slate-blue ribbon; Protein-U, magenta cartoon) and that in *Rba*. *sphaeroides* strain DBCΩG (PDB: 4JC9, red cartoon for PufX, gray ribbons for LH1-αβ). **(b)** Side and top views of superposition of the Cα carbons of the LH1 αβ-polypeptides between the structure in this work (same color scheme as in **(a)**) and that in *Rba*. *veldkampii* PDB 7DDQ (LH1-αβ, gray ribbons; PufX, red cartoon).

a

LH1-α (calculated Mw: 6837.11)

`formyl-MSKFYKIWMIFDPRRVFVAQGVFLFLLAVMIHLILLSTPSYNWLEISAAKYNRVAVAE`

LH1-β (calculated Mw: 5457.12)

`ADKSDLGYTGLTDEQAQELHSVYMSGLWLFSAVAIVAHLAVYIWRPWF`

PufX (calculated Mw: 7459.58)

`ADKTIFNDHLNTNPKTNLRLWVAFQMMKGAGWAGGVFFGTLLLIGFFRVVGRMLPIDENPAPAPNITG`

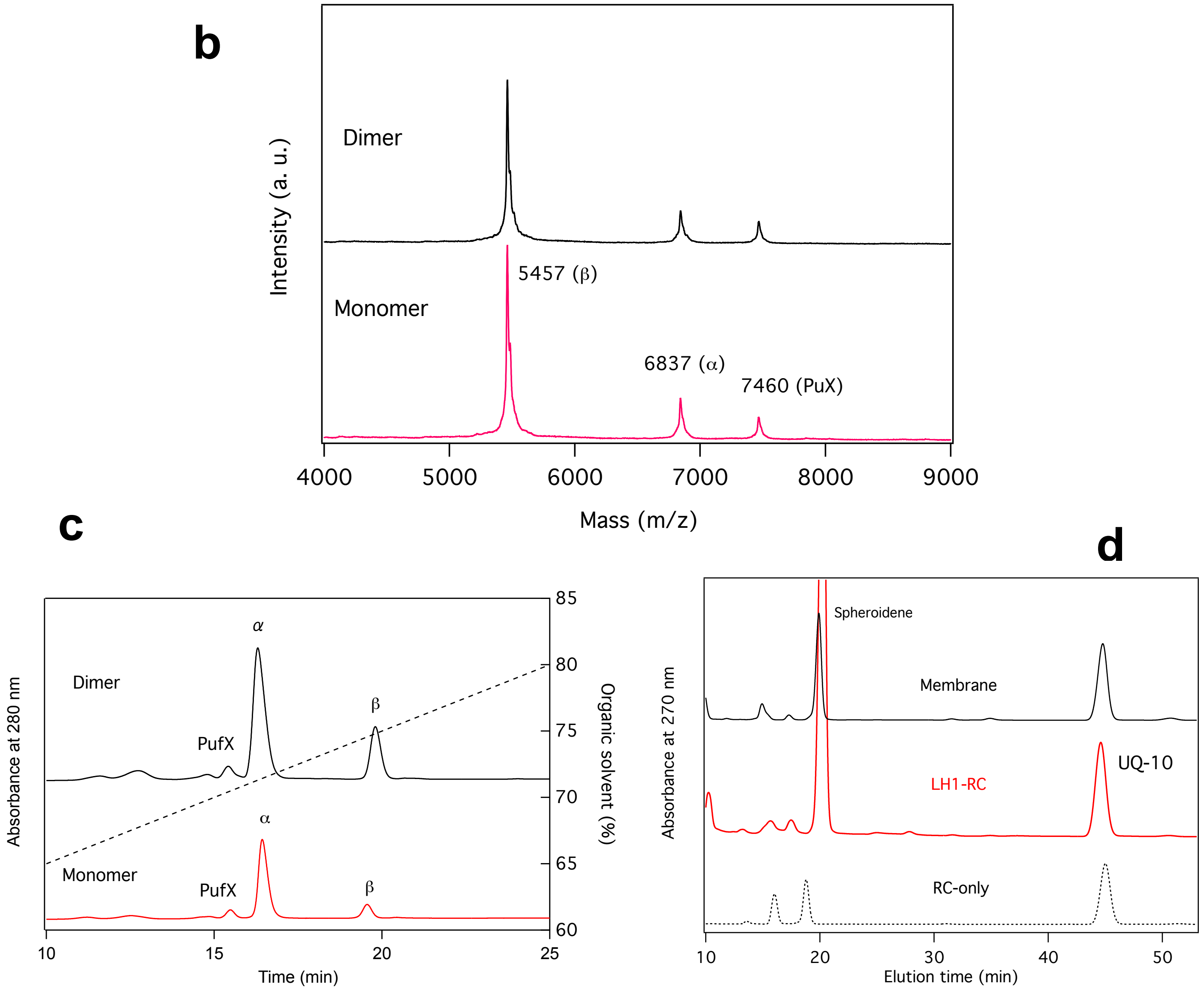

Supplementary Fig. 6 Characterizations of the *Rba. sphaeroides* IL106 LH1-RC complex. (a) Sequences of the expressed LH1-αβ and PufX polypeptides. **(b)** MALDI/TOF-MS spectra of dimeric and monomeric LH1-RCs obtained under the same conditions as described in Ref. 51. **(c)** Reverse-phase HPLC chromatograms (TSKgel, SuperODS, 4.6×100 mm, TOSOH) of the dimeric and monomeric LH1-RCs eluted at 25 ˚C by a gradient of 60–90% organic solvent consisted of acetonitrile/2-propanol (2:1) containing 0.1% trifluoroacetic acid. **(d)** Reverse-phase HPLC chromatograms (TOSOH, TSKgel ODS-80Ts, 4.6×250 mm) of the quinones and pigments from the membranes, purified LH1-RC and RC-only complexes isocratically eluted at 25 ˚C by 7:3 methanol/ isopropanol at flow rate of 0.7 mL/min.

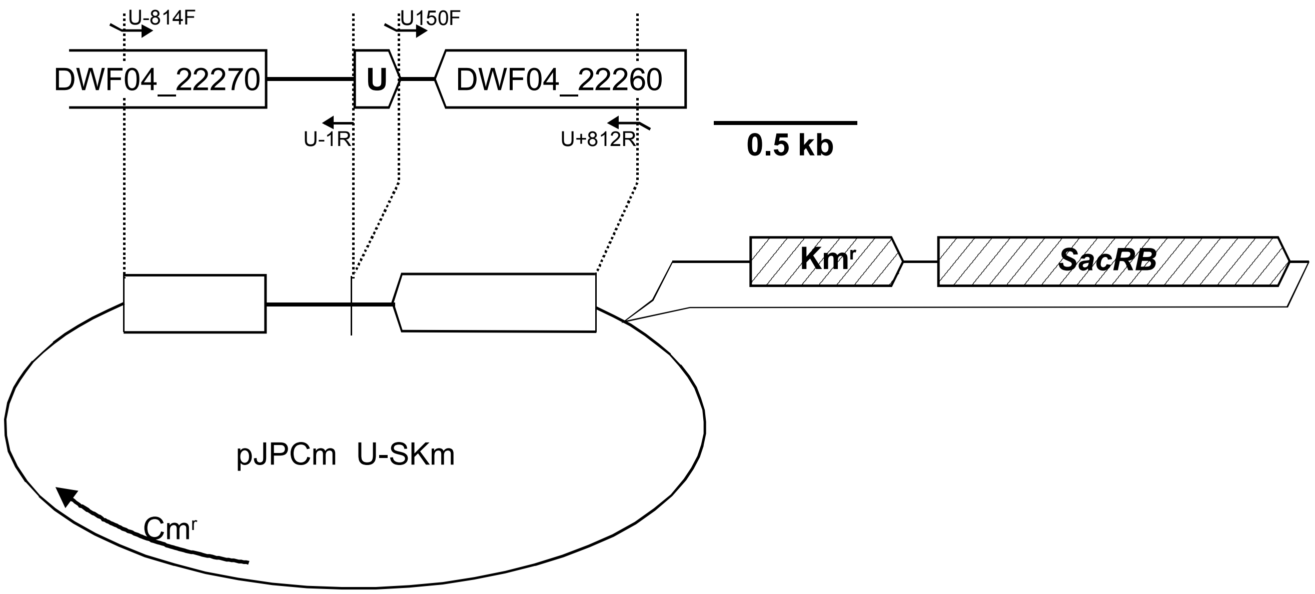

U-814F: 5'-TTGCATGCCTGCAGGTCCACCGCCACGAAGAGGAGGTTG
U-1R: 5'-GGTGCCTCCTTCAGATGCAAGC
U150F: 5'-TCTGAAGGAGGCACCGAACAGCAACTGACGGCACAGC
U+812R: 5'-GGGGATCCTCTAGAGTCGAGTTCACCGACTTCTTCGGCAAC

Supplementary Fig. 7 Schematic representation of gene manipulation. Genes are designated by open boxes with arrow heads showing the direction of transcriptions. The gene encoding the protein-U is labeled by “U”, which is flanked by two ORFs (DWF04_22260 and DWF04_22270 by reference to NCBI database; QRBG01000031) on the complementally strand. Small arrows represent oligonucleotide primers used for PCR. Tabs at the ends of these arrows show additional sequences used for the ligation to the specified DNA fragments. Nucleotide sequences of these PCR primers are shown on the bottom, in which the part of the ligation-tab is underlined.

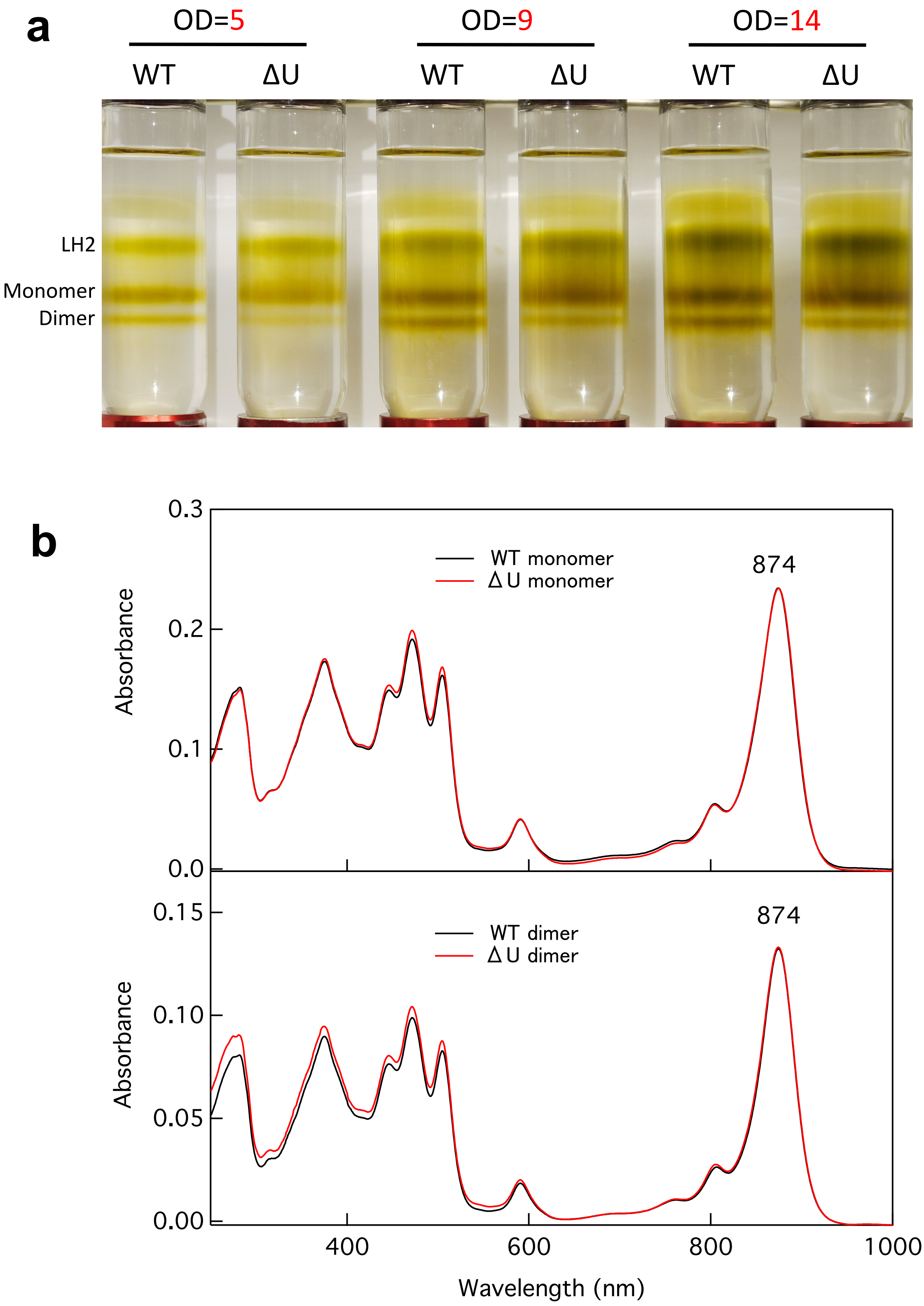

Supplementary Fig. 8 Characterizations of the protein-U-deleted *Rba. sphaeroides* strain IL106ΔU. (a) Sucrose density gradient (10–40% w/v) centrifugations of the solubilized pigment-protein complexes from wild-type (WT) and protein-U-deleted (ΔU) *Rba. sphaeroides* IL106 membranes. 1mL of the solubilized solutions with the concentrations (measured by optical density, OD = 5, 9, 14, at 850 nm) was loaded on the sucrose solution in each tube. **(b)** Normalized absorption spectra of the monomeric (*upper*) and dimeric (*lower*) WT and ΔU LH1-RCs collected from the sucrose density gradient solutions.

a

Species name	Protein-U	PufX	Oligomeric state	References
***Rba. sphaeroides* f. sp. *denitrificans* (IL106)**	**WP_002721225**	**WP_069333028**	**Monomer/Dimer**	*EMBOJ* 1999 18:534
Rba. sphaeroides 2.4.1. (NCIB 8253)	WP_002721225	WP_002720419	Monomer/Dimer	*Nature* 2004 430:1058
Rba. sphaeroides ATCC 17025	WP_085996593	WP_011909039		
Rba. sphaeroides ATCC 17029	WP_002721225	WP_002720419		
Rba. sphaeroides KD131	WP_002721225	WP_002720419		
Rba. sphaeroides WS8N	WP_002721225	WP_002720419		
Rba. azotoformans	WP_085996593	WP_011909039	Monomer/Dimer	*BBA* 2012 1817:336
Rba. johrii	WP_002721225	WP_069333028		
Rba. megalophilus	WP_002721225	WP_002720419		
Rba. ovatus	WP_176504535	WP_097030925		
Rba. sediminicola	WP_085996593	WP_145104437		
Rba. blasticus DSM 2131	not identified	WP_181318217	Monomer/Dimer	*JBC* 2005 280:1426
Rba. capsulatus B6	not identified	WP_013066439	Monomer	*BBA* 2012 1817:336
Rba. veldkampii DSM 11550	not identified	WP_107324827	Monomer	*Struct.* 2007 15:1674
Rba. vinaykumarii	not identified	WP_076363364	Monomer	*BBA* 2012 1817:336
Rba. aestuarii	not identified	WP_076484814		
Rba. flagellatus	not identified	WP_149588102		
Rba. maris	not identified	WP_097069514		
Rba. thermarum	not identified	WP_128514093		
Rba. viridis	not identified	WP_110805120		
Cereibacter changlensis (Rba. changlensis)	not identified	WP_107664449<br>WP_136793197	Monomer/Dimer	*BBA* 2012 1817:336
Rhodobaca bogoriensis LBB1	not identified	WP_071479740	Monomer/Dimer/Trimer	*Phil. Trans. R. Sci.* 2012 367:3412

b

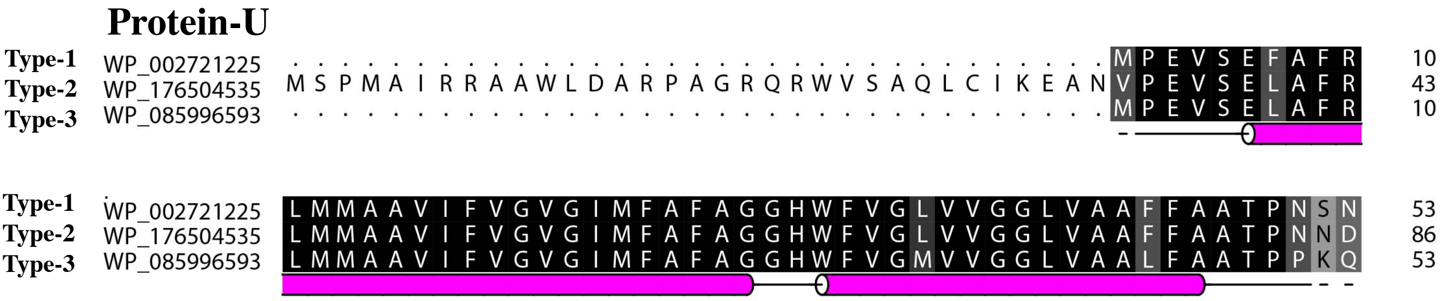

Supplementary Fig. 9 Distributions of the protein-U and PufX in the genus *Rhodobacter*. (a) Distributions of protein-U and PufX (indicated by Protein ID) in the genomes of genus *Rhodobacter*. *Rhodobacter sphaeroides* are shown in red fonts, the *Rhodobacter* species containing protein-U are shown in blue fonts, and other *Rhodobacter* species and *Rhodobaca* without protein-U are shown in black fonts. **(b)** Sequence comparison of protein-U between *Rba. sphaeroides* IL106 (WP_002721225) and others using the CLUSTAL X. The background of residues are colored in gray scale by similarity (Black: identical, White: non-conserved).

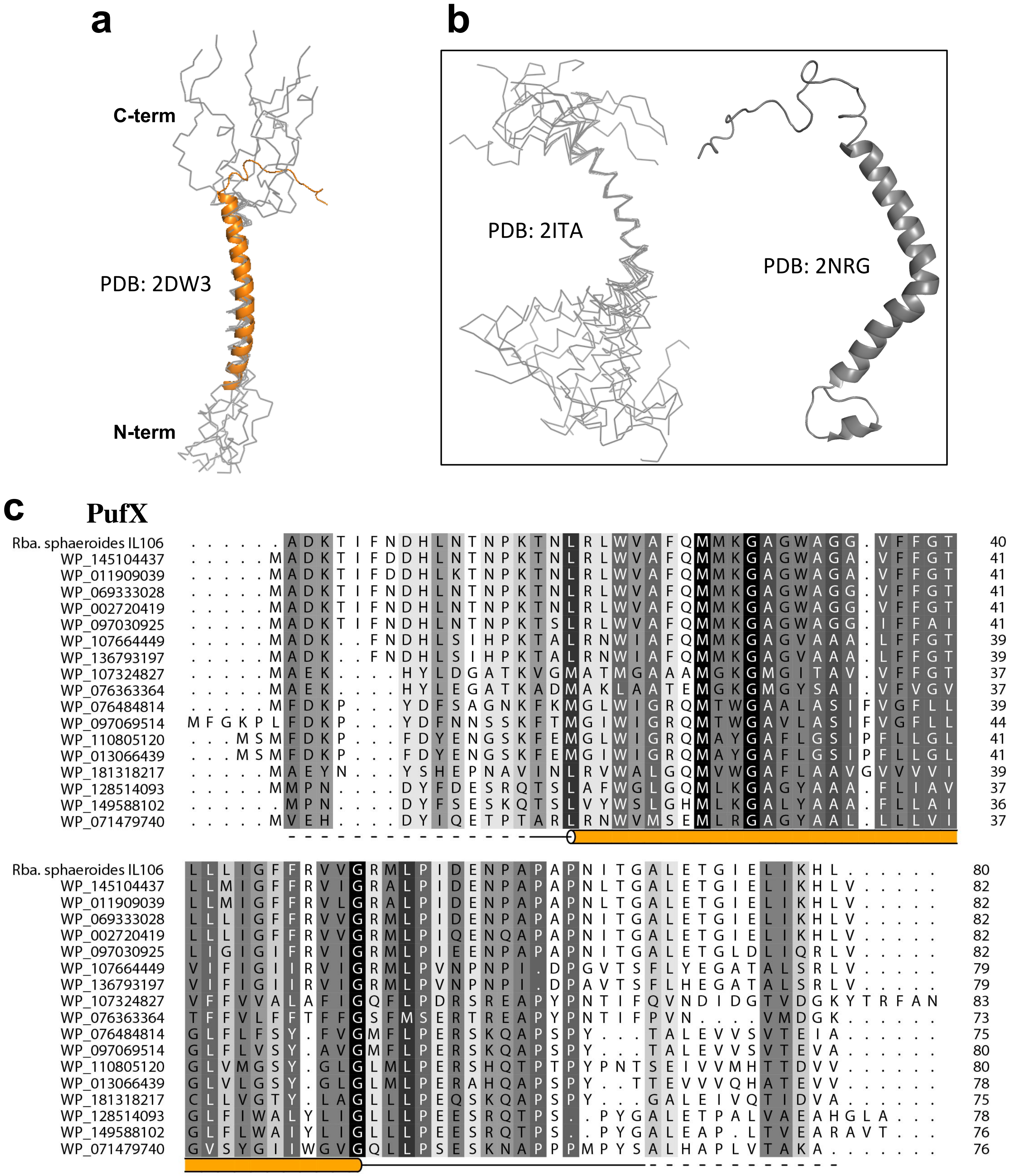

Supplementary Fig. 10 Comparisons of the PufX structures and sequences. (a) Superposition of the transmembrane domains for the *Rba. sphaeroides* PufX structures determined by cryo-EM (this work, colored) and solution NMR (gray ensemble, PDB: 2DW3). **(b)** Alternative solution NMR structure (PDB: 2ITA for the ensemble; PDB: 2NRG for the minimized average) of the *Rba. sphaeroides* PufX polypeptide. **(c)** Sequence alignment of PufX and PufX-like polypeptides using the CLUSTAL X. The background of residues are colored in gray scale by similarity (Black: identical, White: non-conserved).
